# Labour and Landlords

**Published:** 1895-07-20

**Authors:** 


### Social Problems.

### LABOUR AND LANDLORDS.

An interesting little pamphlet by Sir H. Gilzean-Reid,* on the success attending co-operation in housing the people in Edinburgh, draws a forcible contrast between the homes of the workman tenant and the workman house-owner. The picture presented of neat dwellings of three to six apartments, provided with every convenience, the best sanitary arrangements, and a plot of garden ground, might tempt many persons well above the artisan class, when it is found that the whole cost is but £130, payable, if desired, in fourteen yearly instalments. It would, however, be sanguine to expect a very rapid extension of co-operative colonies, depending, as they do, for success on exceptional integrity and thrift, or to look to them, in London at any rate, for a solution of the housing difficulty. The building societies whose operations are carried on round London do, in fact, cater for a different class, build unavoidably at a far higher figure, and depend on other motives besides that of securing for each member a freehold homestead. The majority of members would hold it the height of extravagance to occupy a house acquired much below the market value. To let it to a sound tenant for a good rent is the aim of the saving man, while he and his family live more cheaply elsewhere. It is hardly possible, therefore, that any widespread change in the condition of the workman's dwelling can be attained by co-operation in the teeth of this subtle temptation to sublet a cheaply-acquired house. When all is done, the vast majority will continue to dwell in houses not their own, and, unhappily, under conditions of pressure as regards space which completely nullify the natural and wholesome effects of competition on their surroundings.

Now, dependent as all tenants are on their landlord's goodwill for that wide range of concessions known as "having things done," which go to make up the sum of domestic comfort, the dwellers in tenement houses are in far worse case. They have no control over any part of the house they inhabit except their single room. They may be clean and orderly in their habits, and yet be exposed by the indifference of their landlords at the very threshhold of their door to filthy staircase, dropping plaster, foul smells, and abundant vermin. A street door left wide open till far into the night, with stairs and passage in pitch darkness, they will, of course, expect, and they will not dream of complaining of the riots which make night hideous under these conditions. Yet they will be paying far more per square foot for their miserable travesty of a home than the inhabitants of adjoining mansions with day and night porters, gas lighted staircase, and every modern convenience.

Unhappily all the arrangements of the tenement house are made on the assumption that the tenants are utterly indifferent to cleanliness and home comfort, and this assumption even some of the more respectable are only too ready to justify by adapting themselves with a fatal facility to the low standard of decency around them. From this vicious circle of bad houses arising out of bad tenants, and bad tenants debased by bad houses, who can deliver us? Must the great majority of the poor wait for a healthy home till the philanthropist can find time and money to pull down and reconstruct the myriad tenement houses which are to be found in all our large towns? Down they must come some day, but meantime can nothing be done to improve matters for those who pass their lives and rear their families under these foul and dangerous conditions? Every year infectious diseases break out in these populous quarters, smoulder on in spite of immediate isolation and disinfection, and threaten at last to become endemic in our great cities. And this in the teeth of a whole army of sanitary inspectors, and a system of drainage which is the admiration of the world.

Meantime what seems to be most urgently required is a well-organised system of continual cleansing and repair, enforced by law upon landlord and tenant alike. It is very far from enough to start clean. The dismal interiors of certain "model dwellings" show conclusively the need for incessant renovation. And it does not require much consideration to see how speedily a room occupied night and day by some half-dozen persons will become defiled, nor how dangerous that it should pass many times from one such occupancy to another without efficient cleansing. It ought not to be possible to find, as the writer has done, a family living, eating, sleeping in a room with its door still defiled by tattered scraps of paper used in its disinfection from a fatal case of small-pox four months before, and never cleansed since. If in the registered lodging-house, the factory or workshop, and all places where numbers are massed together, cleanliness is enforced as a safe-guard for themselves and for the community, it is no less imperatively demanded in the crowded tenement house. But as the tenants in these dwellings form a somewhat destructive element, it is reasonable that they should bear their fair share in the continual labour of purification. Upon the landlord should devolve the duty now systematically evaded of keeping in order the common staircase, passage, and offices; he should be compelled to provide for their being washed down daily, and replastered and whitewashed at least four times a year. The compulsory lighting of the common staircase after dark is a reform which cannot now long be delayed. The tenant, on his part, should be required at least once a year to cleanse the paint, repaper or distemper the walls of his room, and whitewash the ceiling, the landlord providing the material, and this process should be enforced on each tenant before leaving. The expense of materials is a mere trifle compared with the cost of labour, and it is one of the mysteries of town life that clever workmen should be content to beautify anyone's home but their own, and should linger for months out of work in rooms literally falling to pieces for want of a few hours' labour.

The history of the last fifty years of successful social legislation has proved that, though dirt and slovenly ways are the natural consequence of herding large numbers of people together, cleanliness is a virtue peculiarly fit to be enforced by law, since those who love it least acknowledge a certain satisfaction in its effects. Dirt is, after all, the great enemy to public health. It has been tracked of late years from one stronghold to another, inspected, disinfected, and banished from public view. It remains now to strike a bold stroke at the common enemy in its last and surest refuge in the homes of the people.

* "Housing the People." By Sir Hugh Gilzean-Reid J.P. (A Gardner.)

